# Preventing sickness absence among employees with common mental disorders or stress-related symptoms at work: a cluster randomised controlled trial of a problem-solving-based intervention conducted by the Occupational Health Services

**DOI:** 10.1136/oemed-2019-106353

**Published:** 2020-04-14

**Authors:** Marijke Keus van de Poll, Lotta Nybergh, Caroline Lornudd, Jan Hagberg, Lennart Bodin, Lydia Kwak, Irene Jensen, Malin Lohela-Karlsson, Margareta Torgén, Gunnar Bergstrom

**Affiliations:** 1 Division of Intervention and Implementation Research in Worker Health, Institute of Environmental Medicine, Karolinska Institute, Stockholm, Sweden; 2 Centre for Musculoskeletal Research, Department of Occupational Health Sciences and Psychology, University of Gävle, Gävle, Sweden; 3 Department of Learning, Informatics, Management and Ethics (LIME), Karolinska Institute, Stockholm, Sweden; 4 Department of Medical Sciences, Uppsala University, Uppsala, Sweden

**Keywords:** intervention studies, OH services, public health, mental health, sickness absence

## Abstract

**Objectives:**

Common mental disorders (CMDs) are among the main causes of sickness absence and can lead to suffering and high costs for individuals, employers and the society. The occupational health service (OHS) can offer work-directed interventions to support employers and employees. The aim of this study was to evaluate the effect on sickness absence and health of a work-directed intervention given by the OHS to employees with CMDs or stress-related symptoms.

**Methods:**

Randomisation was conducted at the OHS consultant level and each consultant was allocated into either giving a brief problem-solving intervention (PSI) or care as usual (CAU). The study group consisted of 100 employees with stress symptoms or CMDs. PSI was highly structured and used a participatory approach, involving both the employee and the employee’s manager. CAU was also work-directed but not based on the same theoretical concepts as PSI. Outcomes were assessed at baseline, at 6 and at 12 months. Primary outcome was registered sickness absence during the 1-year follow-up period. Among the secondary outcomes were self-registered sickness absence, return to work (RTW) and mental health.

**Results:**

A statistical interaction for group × time was found on the primary outcome (p=0.033) and PSI had almost 15 days less sickness absence during follow-up compared with CAU. Concerning the secondary outcomes, PSI showed an earlier partial RTW and the mental health improved in both groups without significant group differences.

**Conclusion:**

PSI was effective in reducing sickness absence which was the primary outcome in this study.

**Key messages**

**What is already known about this subject?**

- Common mental disorders (CMDs) are among the main causes of sickness absence.
- Brief work-directed interventions given by the occupational health service (OHS) to employees with CMDs have shown promising results concerning return to work (RTW).

**What are the new findings?**

- In this cluster randomised study, a participative problem-solving intervention reduced sickness absence among employees with occupational stress or CMDs.
- Symptoms of mental ill-health decreased in both the intervention and control groups during the 12-month follow-up period.

**How might this impact on policy or clinical practice in the foreseeable future?**

- Following a structured and participative problem-solving approach with early involvement of the employer may enhance the effects of work-directed interventions at the OHS.
- Regular follow-ups of RTW plans developed by the OHS, the employee and the employer may facilitate RTW.

## Background

Common mental disorders (CMD, ie, depression, anxiety and adjustment disorders) are among the main causes for (long term) sickness absence in many countries.^1^ CMDs demand medical care consumption and they influence functioning and productivity of the individual. In the EU, one in six people had a mental health problem in 2016,^2^ leading to an estimated total cost of over 600 billion Euros per year whereof indirect costs, such as absenteeism and presenteeism, constitutes the largest part.^2^ Against this background, the need for effective interventions related to work ability is called for.

Studies have found that the combination of high job demands with low levels of control at work can increase the risk for CMD,^3^ while organisational justice and influence at work can decrease the risk.^4 5^ When problems in the work environment contribute to the development of CMD, changes in the work environment (eg, change of responsibilities/assignments; change in work schedule) become important to reduce CMD, and consequently also sickness absence. Interventions or treatments on an individual, group and/or organisational level can help to reduce the risk for continued CMD problems and (new) periods of sickness absence. Adding a work-directed approach to a clinical intervention can be effective in improving work return or decreasing sickness absence.^6^ The aim of work-directed interventions is to facilitate work return, or maintain work ability and/or to help the employee to manage his/her psychiatric symptoms in relation to work.^6^ Several studies compared care as usual (CAU) with work-directed interventions for employees on sick leave due to CMD.^7–12^ In some studies, partial return to work (RTW) was accelerated by the work-directed interventions compared with CAU. However, the results concerning full-time RTW did not show that work-directed intervention was better than CAU.^8 10 11^ Work-directed interventions for improving RTW may also help to prevent future sickness absence for employees that are currently at work but are at risk of future sickness absence due to mild to moderate mental problems or occupational stress.^13^

The occupational health service (OHS) exists in most industrialised countries. It aims to support employers in promoting employee health.^14^ The OHS is also an important actor for rehabilitative measures among employees on sick leave as well as early prevention offered to employees with symptoms of poor health in the workplace.^15^ OHS has knowledge of the employee’s work environment and can offer interventions to prevent sickness absence that take into account both the individual and the workplace.

In this study, we evaluated the effects on sickness absence, RTW and mental health of a work-directed intervention given by the OHS to employees with CMDs or stress-related symptoms at work.^16^ The hypothesis for the primary outcome was that the intervention should reduce sickness absence compared with CAU.

## Methods

### Study design and setting

The study was a two-armed cluster randomised controlled design with a 1 year follow-up period. It was conducted in collaboration with three OHS (four units) in Sweden. The design and procedures of the study has been reported in detail in the study protocol,^16^ but a brief summary will be presented below.

### Inclusion and exclusion criteria

Inclusion criteria were as follows:

- The employee sought help at the OHS for a new episode of occupational stress or symptoms related to CMDs affecting the ability to work. If the employee was on sick leave due to CMDs this period should not have exceeded 3 months.
- The employee should agree to the involvement of the employee’s manager in the intervention.
- The employee had to understand both written and spoken Swedish.

Exclusion criteria were pregnancy, victim of workplace bullying, post-traumatic stress disorder or other severe mental illness or co-morbidity.^16^

### Recruitment of participants

Recruitment occurred between August 2015 and June 2017, in cooperation with the participating OHS. After receiving information about the study and being trained in the recruitment process, consultants recruited employees. The research group contacted the recruited employees to give them more detailed information and to obtain written consent. Further, the employee received the baseline questionnaire, in which receipt of the information about the study had to be confirmed. Study inclusion occurred on returning the completed questionnaire.^16^

### Randomisation

**Figure 1:**
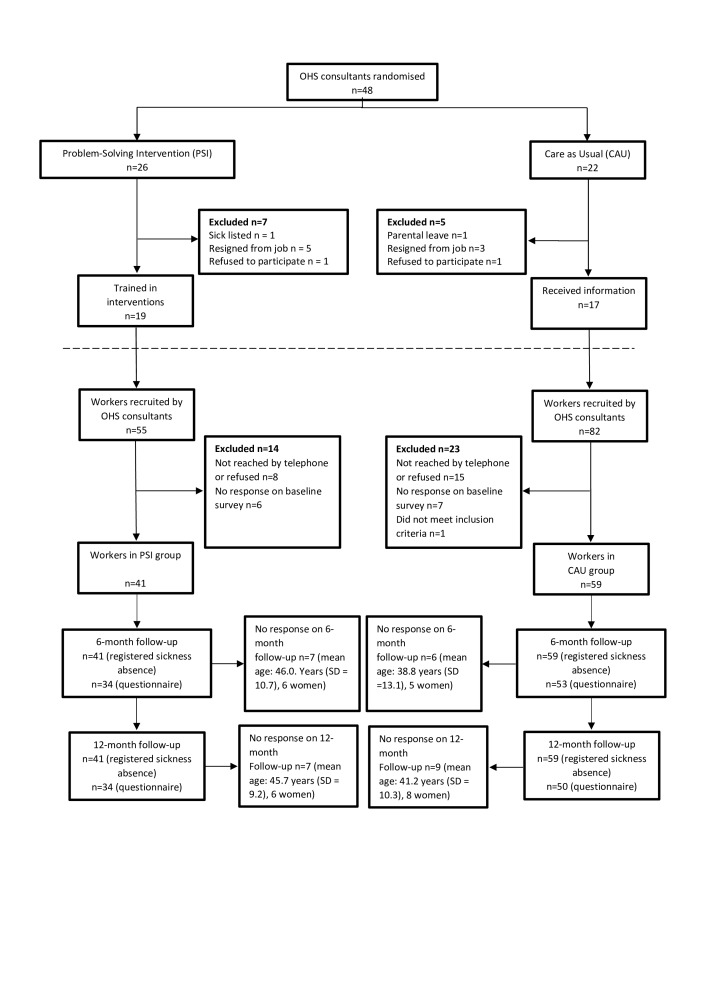
Flow diagram showing the recruitment of consultants, experimental and control groups. OHS, occupational health service.

An independent statistician received lists of eligible OHS consultants and randomised them into the experimental treatment or CAU using computer-generated random numbers (conducted by LB). Randomisation was stratified by OHS unit and we randomised four more consultants to the experimental arm to compensate for recruitment problems at one of the units. See also figure 1, online supplementary table 1 and the protocol.^16^

10.1136/oemed-2019-106353.supp1 Supplementary data

**Table 1:** Employee characteristics per study group at baseline

Sociodemographic characteristics	PSI (n=41)		CAU (n=59)	
Age, years, m (SD)	42.66	(10.39)	44.00	(9.64)
Female, n (%)	37	(90)	43	(73)
Children, n (%)	23	(56)	39	(66)
Education level, n (%)				
Primary/secondary education	14	(34)	20	(34)
Higher education/university	27	(66)	39	(66)
Work-private life balance, m (SD)				
Work influences private life	3.20	(1.23)	3.42	(.91)
Private life influences work	1.98	(0.99)	2.27	(1.05)
Employer, n (%)				
Municipality, county, state*	38	(93)	39	(66)
Private business	3	(7)	20	(34)
Ordinary working hours				
Full-time (40 h/week)	31	(76)	52	(88)
Part-time (<40 h/week)	10	(24)	7	(12)
Presenteeism, n (%)				
No presenteeism	5	(12)	8	(14)
Presenteeism (1 or more times)	36	(88)	51	(86)
HAD, n (%)				
Depression	15	(37)	26	(44)
Mild depression	8	(20)	16	(27)
No depression	18	(44)	17	(29)
Anxiety	20	(49)	26	(44)
Mild anxiety	13	(32)	25	(42)
No anxiety	8	(19)	8	(14)
S-ED, n (%)				
Moderate/pronounced ED	31	(76)	40	(68)
No ED	10	(24)	18	(31)
DCSQ, m (SD)				
Demand	3.04	(0.55)	3.13	(.53)
Control	3.18	(0.36)	2.88	(.43)
Support	3.12	(0.60)	2.95	(.55)
Stress, m (SD)	3.98	(0.89)	4.05	(.90)
Production loss due to ill health, m (SD)	6.20	(2.78)	6.51	(2.37)
Production loss due to work environment problems, m (SD)	6.24	(3.22)	6.64	(3.15)
Registered sickness absence†, n (%)				
No sickness absence	20	(49)	31	(53)
Sickness absence	21	(51)	28	(47)
Diagnoses‡, n (%)				
Reaction to severe stress, and adjustment disorders	17	(41)	24	(41)
Other CMDs§	4	(10)	4	(7)

*Three individuals in PSI and seven employees in CAU were employed by the state.

†In Sweden, sickness absence benefits or disability pension is given for individuals with work-related income for 25%, 50%, 75% or 100% of the employee’s ordinary working hours. The first 14 days of a sickness period, the employer pays approximately 80% of the daily wages of the employee. From day 15 to 90, the employer pays 10% and the SSIA approximately 80%. After 90 days employee compensation is received from the SSIA depending on degree of work ability.^37^

‡Data on diagnoses are obtained from register data from SSIA. Not all participating employees were on sick leave at baseline.

§Other anxiety disorders, single depressive episode and problems related to life management difficulty.

CAU, care as usual; CMD, common mental disorder; DCSQ, Demand Control Support Questionnaire; ED, Exhaustion Disorder; HAD, Hospital Anxiety and Depression scale; PSI, problem-solving intervention; S-ED, self-reported exhaustion disorder; SSIA, Swedish Social Insurance Agency.

### Blinding

Employees were blinded to the possibility of receiving another intervention within the trial. Consultants were not blinded regarding which treatment was given.^16^ The statistician and the researchers were blinded at the initial analysis of the primary outcome but not, due to practical reasons, during the entire process of analyses.

### The problem-solving intervention (PSI)

The OHS consultants received a 1-day training course by members of the research group and a clinical psychologist.^16^ They also received detailed work sheets. The focus of the intervention was primarily on adjusting the work situation and secondarily to give the employee advice concerning stress management. The goal was promotion of work ability and RTW. The theoretical basis for the intervention stems from problem-solving therapy,^12^ and the ‘mismatch’ model concerning the match between the employee and the work environment. It emphasises six aspects of the work situation which are addressed during the meetings (ie, workload, control, reward, community, fairness and values).^17^ In the first two steps of the intervention, the consultant interviewed the manager and the employee, respectively. The third step consisted of a joint meeting in which a participatory approach was applied by which the employee’s manager and the employee were guided by the consultant and encouraged to actively take part in problem solving concerning the work situation.^18 19^ At least three follow-ups of the manager and the employee during a 3-month period were recommended.

### Care as usual

The consultants randomised to CAU received a general introduction in research about psychosocial factors and mental health at work for approximately 1 hour. CAU at the participating OHS implied that both employee and manager usually were involved in the intervention. CAU was neither structured to the same degree as PSI nor based on the same theoretical frameworks.^16^ However, CAU was also work-directed.

### Procedure

Registered data on sickness absence was obtained from the Swedish Social Insurance Agency (SSIA). Questionnaires were administered at baseline, after 6 months and after 12 months. Since the first 14 days of a period of sickness absence (ie, short-term sickness absence) are not covered by the SSIA, SMS messages were sent to the participants every fourth week over the follow-up period, to gather information about all sickness days during the follow-up year, including the first 14 days. SMS as data collection method has shown to be feasible.^20^

### Dependent variables

#### Primary outcome

##### Registered sickness absence

The primary outcome was total number of days of registered sickness absence (sickness benefit and disability pension), defined as the total number of net absence days (all causes) during the 12-month follow-up period.

#### Secondary outcomes

##### Self-reported sickness absence, RTW and production loss

Questions were sent to the participants concerning sick leave and days of sickness absence over the last 4 weeks,^21^ ordinary working hours,^16 22^ production loss due to ill health or work environment problems^23^ and stress^24^ (online supplementary table 2).

10.1136/oemed-2019-106353.supp2 Supplementary data

For those with sickness absence at baseline, partial RTW was calculated as the time from baseline until the employee returned to work in any increased capacity. Full RTW was defined as working ordinary hours over an uninterrupted period of at least 4 weeks.

##### Mental/general health, sleep and work ability

See online supplementary table 3 and the protocol,^16^ for an overview of the questionnaires used to measure depression, anxiety,^25^ exhaustion,^26 27^ general health and sleep problems,^28–30^ presenteeism,^4^ work ability,^31^ job satisfaction^32^ and psychological and social aspects at work.^3 33^

10.1136/oemed-2019-106353.supp3 Supplementary data

### Data analysis

The analyses were conducted as intention-to-treat analyses and run by IBM SPSS statistics V.25.

Baseline variables with a potential impact on the outcome that were deemed to have been unevenly distributed between the experimental and the control condition were added as potential confounders in the analyses. These confounders were considered based on statistical significance and/or clinical significance.

To investigate the primary outcome, registered days of sickness absence, we performed general estimated equations (GEE) using an independent correlation structure and robust variance estimation. Parameter estimates, standard errors and the correlation information criterion were checked for independent and exchangeable correlation structures.

The analyses were conducted in two steps. In step 1, only the outcome variable was analysed concerning its development from baseline and during follow-up to check for potential time, group and time × group effects. In the second step, we checked for potential interaction effects based on gender × group and sickness absence at baseline × group (no sickness absence vs sickness absence).

GEE analyses were also employed to analyse self-reported days on sickness absence, stress and production loss due to ill health or work environment problems.

Cox regression was used to investigate group differences in time to full and partial RTW. In the analyses with Cox regression, only individuals that were on sick leave (part-time or full-time) at baseline were included. Group differences regarding mental health, quality of life, two items of work ability and job satisfaction were analysed with multivariate analysis of variance (ANOVA). We used an alpha of <0.05 to indicate statistical significance.

## Results

Figure 1 shows that 137 employees were recruited (mean=3.7 per consultant) of which 100 were included in the study. The mean age of the excluded employees was 41.2 years and seven were male.

In PSI, most employees (73%) completed the three steps within 6 weeks after baseline. In CAU, 39 employees also had a joint meeting, of which 37 completed it within 6 weeks after baseline.

Table 1 shows the baseline characteristics of the participants in both groups. The distributions of the different baseline characteristics were similar (based on t-test and Mann-Whitney U test) for both groups for all variables except for the variable ‘employer’ (p<0.001), ‘gender’ (p=0.023) and the ‘control’ dimension of the Demand Control Support Questionnaire (p<0.001). These variables were controlled for in the primary analyses.

### Response rate

At 6 months and 12 months after baseline, response rates were 86% and 84%, respectively. In total, 6500 SMS messages were sent to the participants (5 questions × 13 periods × 100 participants). Mean response rate for PSI was 93.6%. For CAU, mean response rate was 90.3%.

### Primary outcome: registered sickness absence

Initially, analyses of disability pension were also planned. However, since none of the participants received disability pension, during the 12-month follow-up, the data only include sickness absence.

**Figure 2:**
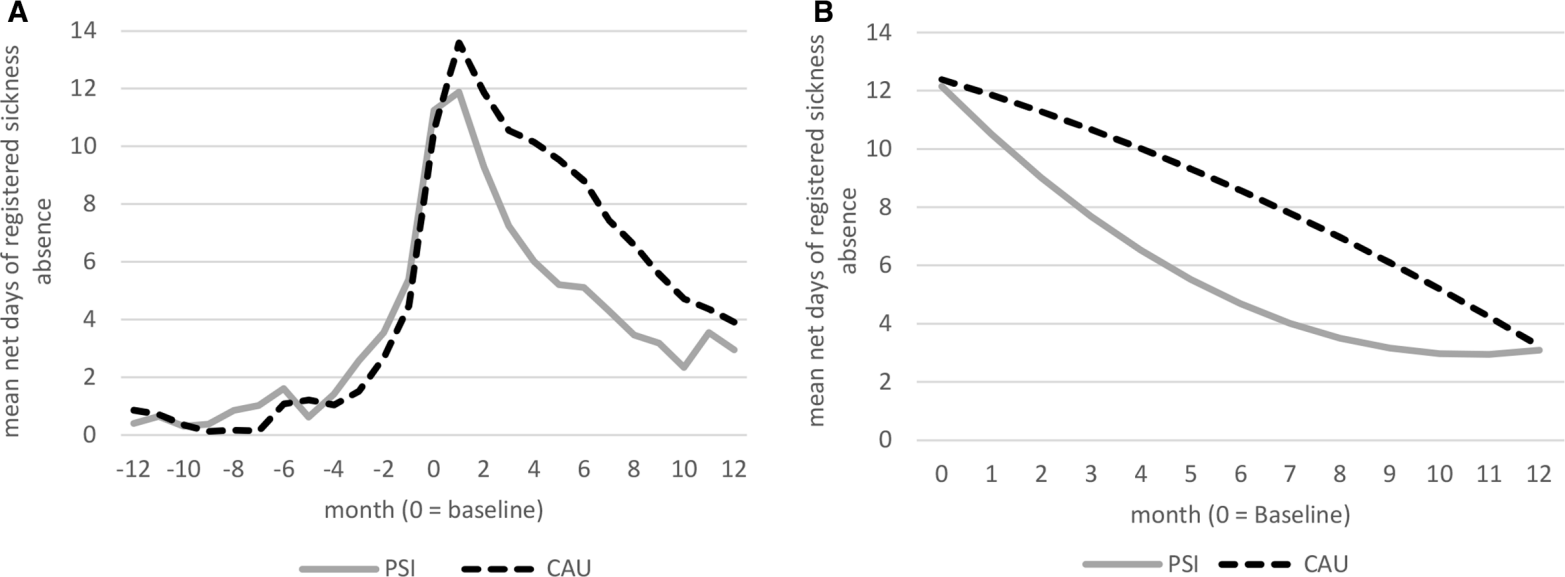
(A) Actual course for registered sickness absence per month 1 year before and 1 year after baseline for PSI and CAU. (B) The course for registered sickness absence per month over the follow-up year for PSI and CAU as estimated by the GEE analysis. Note that the days reported in this figure concern the days that exceed the first 14 days of a sickness absence period, as the first 14 days are not registered by the SSIA. CAU, care as usual; GEE, general estimated equation; PSI, problem-solving intervention; SSIA, Swedish Social Insurance Agency.

Among the employees that received PSI, 15 persons (36.6%) had no registered sickness absence at all during the follow-up. Among the employees receiving CAU, this number was 22 (37.3%). Figure 2A shows the actual course of registered sickness absence over the 12 months before baseline and the follow-up period. Figure 2B depicts estimated second-degree regression curves for the follow-up period for CAU and PSI. Both groups have a similar course of sickness absence until baseline (figure 2A). During the follow-up, PSI has a different course of sickness absence compared with CAU with fairly equal absence at the start and at the end of the period but a pronounced difference at the middle of the period, ie there is a case of statistical interaction between group and time. This interaction revealed to be statistically significant (p=0.033). To give a more detailed picture of the course of sickness absence, table 2 shows the estimated mean differences in sickness absence for each group per month. Statistically significant differences were found for months 5–8. In total, the difference in estimated sickness absence days during these months was almost 15 days, to the advantage of PSI. No interaction for gender × group was found (p=0.242).

**Table 2:** Descriptive figures show mean days and SD for each group per month for sickness absence during the follow-up period (figure 2A). Estimated mean differences in sickness absence for each group per month. The estimated values correspond to the regression model in figure 2B.

Month		Baseline	1	2	3	4	5	6	7	8	9	10	11	12
*Descriptive figures*														
Mean days	PSI	11.3	11.9	9.3	7.2	6.0	5.2	5.1	4.3	3.5	3.2	2.3	3.6	3.0
(SD)		(12.6)	(13.1)	(11.6)	(10.6)	(9.8)	(8.3)	(8.2)	(8.3)	(7.5)	(7.2)	(6.2)	(8.6)	(8.2)
Mean days	CAU	10.6	13.6	11.9	10.6	10.1	9.5	8.8	7.4	6.6	5.6	4.7	4.4	3.9
(SD)		(12.5)	(13.0)	(12.9)	(12.5)	(12.1)	(11.7)	(11.1)	(10.3)	(9.3)	(8.9)	(8.6)	(8.5)	(8.5)
*Estimated Figures*														
Mean days	PSI	12.2	10.5	9.0	7.7	6.5	5.5	4.7	4.0	3.5	3.2	3.0	3.0	3.09
	CAU	12.4	11.9	11.3	10.7	10.0	9.3	8.6	7.8	7.0	6.1	5.2	4.2	3.24
Mean difference		−0.2	−1.4	−2.3	−3.0	−3.5	−3.8	−3.9	−3.8	−3.5	−2.9	−2.2	−1.3	−0.14
95% CI	Lower	−5.4	−5.9	−6.5	−7.0	−7.4	−7.6	−7.6	−7.3	−6.8	−6.1	−5.3	−4.3	−3.38
	Upper	4.9	3.2	1.9	1.0	0.4	0.0	−0.2	−0.2	−0.1	0.3	0.8	1.8	3.10

CAU, care as usual; PSI, problem-solving intervention.

The course of sickness absence during the follow-up differed between employees that were on sick leave at baseline and employees that were not. This interaction between sickness absence at baseline, group and time also revealed to be statistically significant (p<0.001). The same pattern emerged as in the primary analysis; PSI consistently had less sickness absence than CAU regardless of whether the employees were on sick leave or not at baseline (although not statistically significant, reasonably due to the lower statistical power in these analyses that concerned more factors in the model). OHS service was added as a potential confounder, and then the estimated difference between PSI and CAU increased on average by 0.31 days per month during the follow-up period. Employer sector and control at work were unevenly distributed across interventions; however, they did not influence the effect parameter when included as potential confounders in the analytic models.

### Secondary outcomes

#### Self-rated sickness absence, RTW, stress and production loss.

The number of self-reported sickness absence days during the follow-up period was also lower for PSI compared with CAU (online supplementary figure 1), but there was no statistically significant interaction between group and time (p=0.162).

10.1136/oemed-2019-106353.supp4 Supplementary data

A total of 88% in PSI and 76% in CAU had fully returned to work 12 months after baseline, (HR=1.54; 95% CI=0.78; 3.03). PSI had a statistically significant earlier partial RTW compared with CAU (100% partial RTW after 5 respectively 8 months; HR=1.93; 95% CI=1.05; 3.56) (see online supplementary figure 2).

10.1136/oemed-2019-106353.supp5 Supplementary data

For stress, production loss due to ill health and production loss due to work environment problems, there was a statistically significant improvement over time (p=0.001, p<0.001 and p=0.001, respectively), but no statistically significant interactions between group and time (p=0.973, p=0.924 and p=0.462, respectively) were found.

#### Mental/general health, sleep and work ability

According to table 3, the intervention had no statistically significant effect compared with CAU on mental health and stress-related symptoms except for self-perceived general health at 6 months. PSI reported a better general health compared with CAU. There were no statistically significant differences between groups on the ordinal variables self-reported exhaustion disorder (S-ED) and presenteeism, nor for employees’ own prognosis of their work ability in 2 years’ time.

**Table 3:** Mean values (SD) for mental health and stress-related symptoms over time based on raw data. Mean differences between groups calculated with multivariate ANOVA* with baseline as covariate are given in the two columns to the right

	Mean values† (SD)			Baseline 6 months	Baseline 12 months
	Baseline (n=100)	6 months (n=87)	12 months (n=84)	Mean difference (95% CI)	Mean difference (95% CI)
HAD—depression					
PSI	9.00 (4.04)	7.71 (4.44)	6.57 (3.93)	1.14 (−0.53; 2.82)	−0.23 (−1.81; 1.36)
CAU	9.87 (4.61)	7.03 (4.40)	7.04 (3.83)		
HAD—anxiety					
PSI	10.88 (4.31)	9.26 (4.10)	8.79 (4.38)	0.74 (−0.74; 2.21)	0.84 (−0.94;2.62)
CAU	11.22 (3.99)	9.08 (3.80)	7.88 (3.99)		
MBI—exhaustion					
PSI	4.18 (1.54)	3.72 (1.68)	3.55 (1.55)	0.23 (−0.39; 0.84)	<−0.01 (−0.58; 0.58)
CAU	4.23 (1.46)	3.77 (1.68)	3.50 (1.58)		
KSQ sleep quality					
PSI	3.26 (1.37)	3.58 (1.27)	3.62 (1.19)	−0.28 (−0.74;0.18)	−0.11 (−0.56; 0.33)
CAU	3.36 (1.27)	3.86 (1.26)	3.86 (1.22)		
EQ5D					
PSI	71.18 (20.55)	74.34 (19.27)	76.28 (19.01)	−4.46 (−12.68; 3.77)	−1.63 (−9.97; 6.71)
CAU	68.33 (22.49)	76.58 (18.84)	77.62 (20.20)		
Self-perceived general health					
PSI	3.44 (0.90)	3.53 (0.86)	3.29 (0.91)	**0.37 (0.002; 0.73**)	0.18 (−0.18; 0.54)
CAU	3.22 (1.15)	3.11 (0.91)	3.00 (0.90)		
Work ability—physical‡					
PSI	3.63 (1.11)	3.88 (0.91)	4.00 (1.06)	−0.12 (−0.60; 0.37)	−0.11 (−0.52; 0.30)
CAU	3.95 (0.97)	3.83 (1.14)	4.02 (0.94)		
Work ability—psychological‡					
PSI	2.75 (1.03)	3.29 (0.91)	3.75 (0.88)	0.11 (−0.34; 0.55)	−0.23 (−0.62; 0.17)
CAU	2.74 (1.19)	3.34 (1.16)	3.54 (1.09)		
Job satisfaction					
PSI	6.34 (2.12)	6.29 (2.47)	6.88 (1.98)	−0.55 (−1.56; 0.46)	0.15 (−0.79; 1.10)
CAU	6.20 (2.33)	6.52 (2.45)	6.62 (2.57)		

Bold numbers: p<0.05.

*Non-response in multivariate ANOVA was approximately 20% for CAU. It varied between 29% and 22% for PSI.

†The mean values given here are for all participants responding at the follow-ups.

‡Items included in the Work Ability Index.

ANOVA, Analysis of Variance; CAU, care as usual; EQ5D, European Quality of Life 5-Dimensions Questionnaire; HAD, Hospital Anxiety and Depression Scale; KSQ, Karolinska Sleep Questionnaire; MBI, Maslach Burnout Inventory; PSI, problem-solving Intervention.

Statistically significant time effects within both groups were found for depression, anxiety, exhaustion, S-ED, presenteeism and estimation of work ability with respect to psychological demands. See online supplementary tables 4 and 5 for more details.

10.1136/oemed-2019-106353.supp6 Supplementary data

10.1136/oemed-2019-106353.supp7 Supplementary data

## Discussion

The aim of this study was to evaluate the effectiveness of a work-directed intervention given by the OHS to employees with CMDs or stress-related symptoms on sickness absence, RTW and mental health. The primary result showed a statistically significant interaction between time and group, and the estimated net days of registered sickness absence were approximately 15 days lower for PSI than for CAU during follow-up. Furthermore, participants on sickness absence displayed a significantly faster partial RTW for PSI compared with CAU, but no significant differences between groups were detected concerning full RTW. No significant differences were found between PSI and CAU regarding self-reported sickness absence that also included short-term sickness absence. Mental health improved in both groups, but no group differences were found.

Directly after baseline, registered sickness absence increased in both groups, especially in CAU. An explanation might be that high levels of psychological symptoms at baseline caused many participants to take sick leave or to increase the degree of sickness absence.^34^ This may have been subdued by the emphasis on work return or maintaining work in the experimental group.

According to the treatment manual, consultants that delivered PSI should offer at least three follow-up meetings with the employees, preferably during the 12 weeks after the joint meeting. These follow-up meetings might explain the increasing difference in sickness absence between PSI and CAU during the first period of the 12-month follow-up. After the last follow-up meeting, the effect of these meetings might have attenuated which may explain why the difference in sickness absence between PSI and CAU declines during the second part of the 12-month follow-up period. Follow-up meetings were offered in CAU as well, but the content, structure and implementation of these follow-up meetings were not specified. The relation between the follow-up meetings and registered sickness absence will be investigated in a forthcoming process analysis.

The results concerning a faster partial RTW in the PSI group, but no faster full RTW, are in line with earlier studies.^8 10 11 22^ This indicates that PSI facilitates RTW but also that it might benefit from, and be more effective, if it was more comprehensive.

In both groups, symptoms of depression, anxiety and exhaustion were significantly reduced during follow-up, but no differences between groups were found, except for general health. It is not clear to what degree the within-group effects are an effect of treatment or of natural recovery, because all participants received treatment. The level of the symptoms score at the 12-month follow-up indicates that there is clearly room for further improvement both for PSI and CAU. Despite the similar course in symptom reduction for both groups, the PSI group decreased their sickness absence to a higher degree, which appear to support earlier findings that psychological symptoms and RTW are at least partially independent from each other and that work return need to be addressed specifically.^8 35 36^

Since sickness absence due to CMDs is a major challenge in many countries,^1^ the results should be of interest from an international perspective. Both the OHS perspective and the content and design of the intervention were inspired by earlier work from the Netherlands.^8 10 35^ Since our results in general are in line with results from these studies, there appear to be some generalisability across countries and OHS contexts.^14 15^

### Methodological considerations

A strength of this study is the randomised controlled design and the blinding of participants concerning the treatment condition they adhered to. Furthermore, the primary outcome sickness absence was gathered from public registers and the response rates on the self-report measurements were high. Further, the participants were recruited from OHS across different parts of Sweden to primarily enhance the generalisability of the results to the Swedish context.

In table 2, where the course of the primary outcome sick leave days during the follow-up period was specified, multiple significance tests were done, and these results should be interpreted with caution due to the risk of type I error.

The study did not reach the planned size of the population, which was 150 participants. This brings a lack of statistical power and increases the risk of type II errors and the risk that actual differences between PSI and CAU are missed out. Since 80% of the study population consisted of women it is also unclear if the results are relevant for men.

The study was originally designed to include only employees on sickness absence due to CMDs; however, the recruitment became very slow by using this criterion. Consequently, to be able to finish the study within the planned time frame, the inclusion criteria were widened also to accept employees still at work who sought help for stress symptoms or CMDs at the OHS. As mentioned in the results, PSI descriptively showed less days on sick leave compared with CAU also among those not on sick leave at baseline; however, statistical analyses were not meaningful to do due to lack of statistical power. Furthermore, a larger number of participants were recruited to CAU than to PSI. Due to organisational reasons at one of the participating OHS the consultants were not given some extra resources at the start of the study they were asking for. Therefore, very few employees were recruited by these consultants. This issue was not evident at any of the other three OHS units. Finally, the risk of selection bias cannot be excluded as it was the consultants that invited employees for the study.

Consultants in CAU received a 1 hour lecture on psychosocial factors and mental health and this was not considered to have any important impact on the content of the CAU intervention since it did not include any specific techniques, or recommendations, on how to decrease sickness absence among employees. The focus in this report is on registered sickness absence. The secondary analyses are explorative and a complement to the primary analyses. For that reason, we have not discussed all secondary outcomes in detail.

### Implications

There has been some evidence that work-directed interventions with problem-solving strategies are effective in promoting RTW and in decreasing the length of sickness absence. This study expands this evidence as it indicates that a work-directed intervention with a problem-solving strategy is more effective compared with CAU in decreasing sickness absence among employees with CMDs or stress-related symptoms at work. A reduction in sickness absence may also reduce costs for the employer, the employee and society at large. A forthcoming study will explore the cost-effectiveness of the intervention and a process analysis will establish more details about the implementation of the intervention at the OHS.
