# Supplementary material for: Preventing sickness absence among employees with common mental disorders or stress-related symptoms at work: a cluster randomised controlled trial of a problem-solving-based intervention conducted by the Occupational Health Services
Source: Occup Environ Med. 2020 Apr 14;77(7):454–61. doi: 10.1136/oemed-2019-106353 (PMC7306872; doi:10.1136/oemed-2019-106353)
Supplement: Supplementary data [file oemed-2019-106353supp001.pdf]

Supplemental Table 1. Profession and gender for the OHS consultants

	PSI<br>n (%)	CAU<br>n (%)
Female	21	19
Male	5	3
Nurses and ergonomists	16	17
Behavioral scientists and psychologists	8	4
Physician	2	1

PSI = Problem-Solving Intervention; CAU = Care As Usual
In Sweden, the employer pays for the Occupational Health Services. Just below 60% of all employees in Sweden have access to OHS.
