# Supplementary material for: Preventing sickness absence among employees with common mental disorders or stress-related symptoms at work: a cluster randomised controlled trial of a problem-solving-based intervention conducted by the Occupational Health Services
Source: Occup Environ Med. 2020 Apr 14;77(7):454–61. doi: 10.1136/oemed-2019-106353 (PMC7306872; doi:10.1136/oemed-2019-106353)
Supplement: Supplementary data [file oemed-2019-106353supp002.pdf]

Supplemental Table 2. SMS messages were sent to the participants every fourth week over the follow-up period with start one month after the baseline questionnaire was completed.

**Question**		**Response format**
**1**	Are you currently on sick leave?	no sick leave, 25%, 50%, 75% or 100% sick leave
**2**	Over the last eight weeks, have you worked your ordinary working hours for a consecutive period of four weeks?	yes or no. This item was developed for this study.
**3**	Over the last four weeks, how much did your health problems affect your performance while you were working?	0 to 10; higher numbers indicate higher performance limitations
**4**	How many days over the last four weeks have you been on sickness absence (part- or full-time)?	0 – 28 days
**5a**	Have you been stressed lately?	1 to 5; higher values indicate more stress
**5b**	Over the last four weeks, how much did work environment problems affect your performance while you were working?	0 to 10, higher numbers indicate higher performance limitations

Question 5a and 5b were alternated, so each question was asked every other four week-period.
