# Supplementary material for: Preventing sickness absence among employees with common mental disorders or stress-related symptoms at work: a cluster randomised controlled trial of a problem-solving-based intervention conducted by the Occupational Health Services
Source: Occup Environ Med. 2020 Apr 14;77(7):454–61. doi: 10.1136/oemed-2019-106353 (PMC7306872; doi:10.1136/oemed-2019-106353)
Supplement: Supplementary data [file oemed-2019-106353supp003.pdf]

Supplemental Table 3. Questionnaires used to measure secondary outcomes

Measure	*Questionnaire*	*Rating*
Depression and anxiety [25]	Hospital Anxiety and Depression Scale (HAD)	Index score <8: no depression; 8-10: mild depression; index score >10: depression
Exhaustion [26]	The exhaustion scale in the Maslach Burnout Inventory – General Survey (MBI-GS).	7-point scale (0 = never; 6 = every day)
Stress-related exhaustion disorder [27]	Self-reported exhaustion disorder (s-ED) questionnaire	"no", "mild" or "severe"
Sleep problems [28]	Insomnia index in the Karolinska Sleep Questionnaire (KSQ).	6-point scale (0 = always; 6 = never)
Health related quality of life [29]	European Quality of Life – 5 Dimensions questionnaire (EQ5D-3L).	Index with interval 0 to 100; higher scores indicate better health
Self-perceived general health [30]	Single item	5-point scale (1 = excellent; 5 = very poor)
Presenteeism [4]	Single item	4-point scale (1 = never; 4 = more than five times (over the last six months))
Work ability [31]	Three items from the Work Ability Index (WAI)[1]	5-point scale (1= very bad; 5= very good) and 3-point scale (1 = probably not; 3 = probably yes)
Job satisfaction [32]	Single item	11-point scale (0 = dissatisfied; 10 = totally satisfied.
Psychological and social aspects at work [3, 33]	Demand Control Support Questionnaire (DCSQ)	Indexes for demand, control and support: higher values indicate higher demands, control and support
	Parts from the General Nordic Questionnaire (QPS Nordic)	4-point scale (1 = not at all; 4 = a lot) and 5-point scale (1 = seldom or never; 5 = (almost) always)

[1] The items concern the employees' own prognosis of their work ability in 2 years' time (i.e. future work ability) and their work ability in relation to the physical and psychological demands of the job (i.e. work capacity-physical and work capacity-psychological);
