# Supplementary material for: Preventing sickness absence among employees with common mental disorders or stress-related symptoms at work: a cluster randomised controlled trial of a problem-solving-based intervention conducted by the Occupational Health Services
Source: Occup Environ Med. 2020 Apr 14;77(7):454–61. doi: 10.1136/oemed-2019-106353 (PMC7306872; doi:10.1136/oemed-2019-106353)
Supplement: Supplementary data [file oemed-2019-106353supp004.pdf]

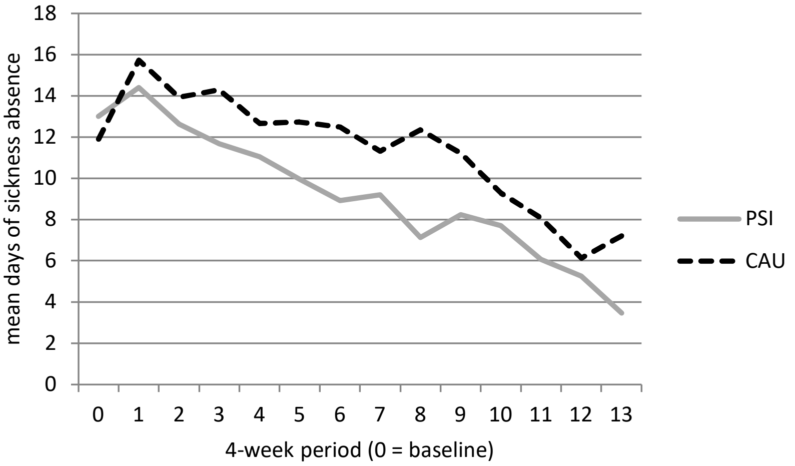

Supplemental Figure 1. Mean number of days of sickness absence per month for the self-registered sickness absence for the one-year follow-up period. Note that this data includes both short-term (up to 14 days) and self-registered long-term sickness absence. PSI = Problem-Solving Intervention; CAU = Care As Usual
