# Supplementary material for: Preventing sickness absence among employees with common mental disorders or stress-related symptoms at work: a cluster randomised controlled trial of a problem-solving-based intervention conducted by the Occupational Health Services
Source: Occup Environ Med. 2020 Apr 14;77(7):454–61. doi: 10.1136/oemed-2019-106353 (PMC7306872; doi:10.1136/oemed-2019-106353)
Supplement: Supplementary data [file oemed-2019-106353supp005.pdf]

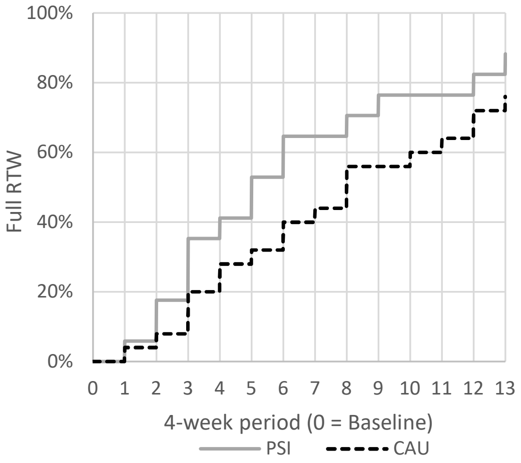

a

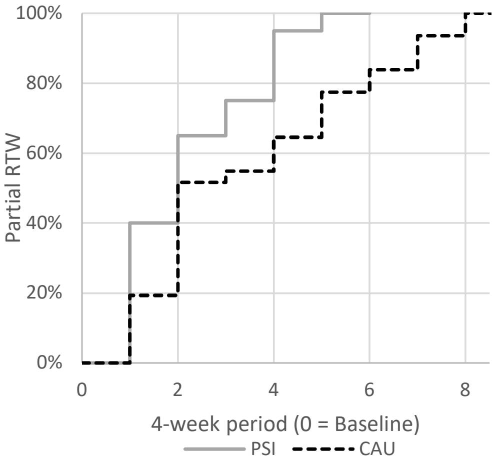

b

Supplemental Figure 2. (a) Kaplan-Meier curve showing the part of the groups that had fully returned to work measured over the 12-month follow-up period. (b) Kaplan-Meier curve showing the part of the groups that partially returned to work measured over the 12-month follow-up period. PSI = Problem-Solving Intervention; CAU = Care As Usual
