# Supplementary material for: Preventing sickness absence among employees with common mental disorders or stress-related symptoms at work: a cluster randomised controlled trial of a problem-solving-based intervention conducted by the Occupational Health Services
Source: Occup Environ Med. 2020 Apr 14;77(7):454–61. doi: 10.1136/oemed-2019-106353 (PMC7306872; doi:10.1136/oemed-2019-106353)
Supplement: Supplementary data [file oemed-2019-106353supp006.pdf]

Supplemental Table 4. Paired t-test for baseline compared to 6 resp. 12 months for PSI and CAU.

		Baseline-6 months		Baseline-12 months	
		**Mean difference**	**95% CI (lower; upper)**	**Mean difference**	**95% CI (lower; upper)**
Depression					
	PSI	1.88	(0.77; 3.00)	3.01	(1.56; 4.47)
	CAU	3.02	(1.82; 4.22)	3.01	(1.78; 4.24)
Anxiety					
	PSI	1.62	(0.56; 2.68)	2.26	(0.54; 3.99)
	CAU	2.21	(1.05; 3.36)	3.20	(1.92; 4.48)
Exhaustion					
	PSI	0.39	(0.03; 0.75)	0.75	(0.22; 1.28)
	CAU	0.58	(0.17; 1.00)	0.70	(0.33; 1.07)
KSQ					
	PSI	-0.34	(-0.71; 0.04)	- 0.44	(-0.82; -0.07)
	CAU	-0.52	(-0.82; -0.23)	- 0.51	(-0.83; -0.19)
EQ5D					
	PSI	-3.97	(-11.48; 3.54)	-6.93	(-15.59; 1.74)
	CAU	-9.66	(-16.29; -3.02)	-10.31	(-16.27; -4.35)
Self-perceived general health					
	PSI	-0.09	(-0.38; 0.20)	0.15	(-0.18; 0.47)
	CAU	0.11	(-0.21; 0.43)	0.12	(-0.18; 0.42)
Work capacity-physical[1]					
	PSI	-0.24	(-0.62; 0.15)	-0.52	(-0.90; -0.13)
	CAU	0.02	(-0.35; 0.38)	-0.18	(-0.47; 0.11)
Work capacity-psychological[1]					
	PSI	-0.52	(-0.90; -0.13)	-1.06	(-1.41; -0.72)
	CAU	-0.71	(-1.00; -0.41)	-0.84	(-1.13; -0.54)
Job satisfaction					
	PSI	0.21	(-0.54; 0.95)	- 0.55	(-1.30; 0.21)
	CAU	-0.37	(-1.11; 0.38)	- 0.42	(-1.08; 0.24)

PSI = Problem-Solving Intervention; CAU = Care As Usual; KSQ = Karolinska Sleep Questionnaire; EQ5D = European Quality of Life 5-Dimensions questionnaire;
[1]Items from the Work Ability Index regarding work ability in relation to the physical and psychological demands of the job
