# Supplementary material for: Preventing sickness absence among employees with common mental disorders or stress-related symptoms at work: a cluster randomised controlled trial of a problem-solving-based intervention conducted by the Occupational Health Services
Source: Occup Environ Med. 2020 Apr 14;77(7):454–61. doi: 10.1136/oemed-2019-106353 (PMC7306872; doi:10.1136/oemed-2019-106353)
Supplement: Supplementary data [file oemed-2019-106353supp007.pdf]

Supplemental Table 5. Wilcoxon Signed-Rank and Mann-Whitney U test for baseline compared to 6 resp. 12 months for PSI and CAU.

	Baseline – 6 months		Baseline-12 months	
	Z	p	Z	p
S-ED				
PSI	-1.41	0.157	-3.64	<.001
CAU	-3.43	0.001	-3.51	<.001
Between groups	-1.26	0.206	-1.34	0.182
Future work ability[1]				
PSI	<.001	1.000	-2.49	0.013
CAU	-0.19	0.847	-0.21	0.833
Between groups	0.07	0.942	-1.80	0.071
Presenteeism				
PSI	-2.99	0.003	-2.36	0.018
CAU	-3.27	0.001	-3.19	0.001
Between groups	0.31	0.759	0.31	0.755

PSI = Problem-Solving Intervention; CAU = Care As Usual; S-ED = Self-reported Exhaustion Disorder;
[1]Item from the Work Ability Index regarding employees' own prognosis of their work ability in two years' time.
